# Social isolation and cognitive decline in older adults: a longitudinal study across 24 countries

**DOI:** 10.1186/s12877-025-06430-6

**Published:** 2025-10-14

**Authors:** Wang Zhang, Jingjing Zhang, Na Gao

**Affiliations:** School of Humanities, Southeast University, Nanjing, Jiangsu 211189 China

**Keywords:** Social isolation, Cognitive ability, Cognitive decline, Cross-National study, Longitudinal study

## Abstract

**Background:**

Cognitive decline represents a grave public health concern associated with aging, and its prevalence has been demonstrated to be associated with elevated rates of disability, dementia risk, and mortality. In recent years, social isolation has emerged as a significant social determinant that may exacerbate cognitive deterioration in older adults.

**Methods:**

Drawing on harmonized data from five major longitudinal aging studies across 24 countries (*N* = 101,581), we constructed standardized indices to assess social isolation and cognitive ability. Linear mixed models and multinational meta-analyses were employed to examine their association. To address potential endogeneity and reverse causality, we further applied the System Generalized Method of Moments (System GMM), leveraging lagged cognitive outcomes as instruments to more robustly identify dynamic relationships. Finally, multilevel modeling and interaction analyses were used to investigate moderating effects at both the country level (e.g., GDP, income inequality, welfare systems) and the individual level (e.g., gender, socioeconomic status, age).

**Results:**

Social isolation was significantly associated with reduced cognitive ability (pooled effect = -0.07, 95% CI = -0.08, -0.05), with consistently negative effects across memory, orientation, and executive ability. System GMM analyses supported these findings and mitigated endogeneity concerns (pooled effect = -0.44, 95% CI = -0.58, -0.30). Cross-nationally, stronger welfare systems and higher levels of economic development buffered the adverse effects, while impacts were more pronounced in vulnerable groups, including the oldest-old, women, and those with lower socioeconomic status.

**Conclusions:**

Our findings underscore the need for cross-national interventions that strengthen social support, increase opportunities for social participation, improve welfare provisions, and foster social integration to mitigate the cognitive health risks posed by social isolation, thereby promoting healthy aging globally.

**Supplementary Information:**

The online version contains supplementary material available at 10.1186/s12877-025-06430-6.

## Background

Cognitive decline has emerged as a salient public health concern amid global population aging. It not only undermines older adults’ autonomy in daily activities but also significantly elevates the risk of dementia. According to the 2021 Global Burden of Disease Study (GBD), cognitive impairment ranks among the leading risk factors for disability and mortality in older populations worldwide, with projections estimating that the global dementia population will surpass 150 million by 2050 [1, 2]. In rapidly aging societies such as the United States, Europe, China, and Japan, the number of individuals with cognitive impairment is increasing sharply, imposing considerable pressure on local healthcare systems and socio-economic development. For instance, the number of dementia cases in China is expected to reach 47.8 million by 2060 [3], while European countries face escalating dementia care costs, underscoring the urgent need to elucidate the social determinants of cognitive impairment [4].

Social isolation, as a structural risk factor with profound implications for the physical and mental health of older adults, has recently garnered growing scholarly attention in the field of cognitive health [5–8]. Rooted in traditions of social capital theory and social network analysis, it is defined as a condition marked by limited social ties, sparse interpersonal networks, and infrequent social interactions [9]. This condition may accelerate cognitive decline in later life via psychological, physiological, and social mechanisms [10]. From a physiological perspective, neuroplasticity theory suggests that prolonged lack of social interaction can reduce cognitive stimulation, diminish neural activity, and contribute to neurodegenerative changes such as brain atrophy and synaptic loss [11]. Longitudinal evidence further indicates that older adults experiencing chronic social isolation are at heightened risk of developing pathological cognitive impairments over time [12, 13]. Psychologically, social isolation is often accompanied by negative emotional states—such as loneliness, chronic stress, and depression—which may induce neuroinflammation and elevate cortisol levels, ultimately leading to neural injury and impaired cognitive functioning [14, 15]. From a broader social capital perspective, isolation limits individuals’ access to social resources and acts as a distal determinant of cognitive health. It affects the accumulation and maintenance of cognitive reserve and influences downstream pathways including neural integrity, health behaviors, and cognitive aging [16]. Cross-national studies have shown that in Nordic countries with strong social capital and community infrastructure, the detrimental impact of isolation on cognition is substantially buffered [17]. Building on this theoretical foundation, the present study explores how social isolation may accelerate cognitive aging among older adults by depleting cognitive reserve, thereby extending existing frameworks in both theory and evidence.

While prior research has shed light on the potential detrimental effects of social isolation on cognitive health, several critical questions remain. First, a clear theoretical consensus has yet to emerge regarding the directionality of the relationship between social isolation and cognitive decline. Some research suggests that isolation precedes cognitive deterioration by limiting cognitive stimulation and impairing neuroplasticity [6]. Conversely, other studies highlight that cognitive decline may reduce individuals’ capacity for social engagement, thereby intensifying their isolation, suggesting a bidirectional or reverse causal relationship [18–20]. This study addresses this complexity by employing long-term panel data and System GMM estimation to mitigate endogeneity and account for lagged effects. Second, while the adverse cognitive effects of social isolation have been observed in multiple national contexts, their variability across cultural settings remains underexplored. For instance, in many Asian societies, limited social participation among older adults is often offset by strong family-based support networks, which may buffer the cognitive risks of isolation [21, 22]. Conversely, in more individualistic societies such as those in North America and Europe, social atomization and prevalent solo living may exacerbate isolation’s cognitive consequences [23]. To this end, the study examines how institutional environments and cultural norms moderate these relationships, thereby informing more context-sensitive intervention strategies. Furthermore, existing studies have paid insufficient attention to the heterogeneous effects of social isolation across different demographic subgroups, particularly in relation to cognitive vulnerability linked to socioeconomic status, gender, and age. The health impacts of isolation are far from uniform; rather, they are shaped by stratified access to resources, divergent social role expectations, and varying capacities to cope with social adversity [24, 25]. Guided by the frameworks of social capital and social embeddedness theory, this study posits that disparities in resource accessibility not only influence individuals’ resilience against isolation but also determine the sustainability and recoverability of their cognitive reserve. Under certain conditions, such disparities may exacerbate structural vulnerabilities and profoundly affect cognitive aging trajectories [26]. Recognizing and unpacking such heterogeneity is therefore essential not only for advancing theoretical insights but also for shaping equitable and just policy responses in public health.

To address the aforementioned research gaps and empirical demands, this study draws upon Ecological Systems Theory and Social Embeddedness Theory to systematically examine the long-term dynamic impact of social isolation on cognitive ability in older adults. Ecological Systems Theory, developed by Bronfenbrenner, conceptualizes individual cognitive development as embedded within a multilayered and interacting set of social contexts—from the microsystem of familial ties, through the mesosystem of neighborhood and community engagement, to the broader exosystem and macrosystem of institutional and cultural structures. Particular emphasis is placed on how the interaction between meso- and exo-level systems shapes the formation and preservation of cognitive reserve [27]. Complementarily, Social Embeddedness Theory, initially articulated by Polanyi and further advanced by Granovetter, argues that individual behavior is deeply rooted in social networks [28]. Its application in medical sociology and aging research has significantly enhanced our understanding of how macro-structural factors influence individual health outcomes, including cognitive health [29, 30].

Methodologically, this study constructs a dynamic, cross-national analytical framework based on longitudinal aging cohort data from 24 countries, combining linear mixed-effects models and system generalized method of moments (System GMM). The former captures both within-individual changes over time and between-group structural differences, while the latter addresses potential bidirectional relationships and unobserved individual heterogeneity, thereby mitigating endogeneity and fixed-effect biases. This approach enhances the robustness of causal inference regarding the impact of social isolation on cognitive ability over time. The study pursues three key objectives: (1) to examine the dynamic influence of social isolation on overall cognitive ability and its specific domains; (2) to assess how national-level socioeconomic structures moderate this relationship; and (3) to explore the heterogeneous effects of social isolation across demographic subgroups. Through this multidimensional empirical strategy, the study aims to enrich theoretical understanding at both the levels of generalizability and heterogeneity while informing precision-targeted policy interventions. In the context of accelerating global aging, the erosion of traditional family support systems, and the social restructuring triggered by the COVID-19 pandemic, clarifying both the universal mechanisms and contextual contingencies of social isolation’s impact on cognitive health offers critical evidence for the development of targeted, context-sensitive interventions. This has far-reaching implications for alleviating the global burden of cognitive decline and promoting healthy aging worldwide.

## Methods

### Data

To facilitate cross-national comparisons of aging issues, this study utilizes the Global Gateway to Aging Data provided by the USC Global Research Network on Aging and Health Policy [31, 32]. Based on three core dimensions—geographical coverage, heterogeneity of aging stages, and socio-economic gradient—five representative national aging surveys were selected: the China Health and Retirement Longitudinal Study (CHARLS), the Korean Longitudinal Study of Aging (KLoSA), the Mexican Health and Aging Study (MHAS), the Survey of Health, Ageing and Retirement in Europe (SHARE), and the Health and Retirement Study (HRS). These datasets cover 24 countries worldwide, constituting a cross-cultural comparative framework encompassing East Asia, North America, Europe, and Latin America. All datasets adopt a longitudinal design, effectively capturing various changes exhibited by older adults over long-term follow-ups.

To minimize the influence of cohort effects and ensure temporal consistency across countries, this study implements a “temporal harmonization strategy” informed by existing research, aiming to establish a unified timeline framework to enhance cross-national comparability and analytical rigor. The data processing involved three key steps: (1) Following the WHO’s definition of older adults (aged ≥ 60), target samples were consistently selected from each national cohort; (2) Missing values in baseline social isolation indicators and core covariates were handled using listwise deletion to ensure complete and consistent measurement; (3) To improve the robustness of longitudinal analyses, only respondents with at least two rounds of cognitive assessments were retained. Detailed procedures are presented in Extended Data Fig. 1. Ultimately, CHARLS included five waves from 2011 to 2020 (average interval of 2–3 years); KLoSA included six waves from 2010 to 2020 (every 2 years); MHAS covered three waves in 2012, 2015, and 2019 (average interval of 3 years); SHARE spanned five waves from 2010 to 2020 (approximately every 2 years); and HRS comprised six waves from 2010 to 2022 (biennially). In total, 101,581 older adults were included, yielding 208,204 observations, and forming a cross-national dynamic cohort with an average follow-up duration of 6.0 years (interquartile range: 4.0–6.0).

### Measures

#### Social isolation and cognitive ability

Both social isolation and cognitive ability were treated as time-varying variables to capture the dynamic changes in social engagement and cognitive health among older adults. For social isolation, this study adopts a multidimensional framework of structural social isolation, drawing on the internationally recognized social network theory by Berkman and Syme [33–35]. Specifically, four objective indicators were used: marital status, living arrangement, family interaction, and social participation. Respondents were asked four key questions regarding their work status: whether they were unmarried (including separated, divorced, or widowed), whether they lived alone, whether they had contact with their children less than once a week, and whether they have not participated in any social activities in the past month. A positive response to any of these questions was considered indicative of social isolation. A social isolation index (ranging from 0 to 4) was constructed by summing these four binary variables, with higher scores indicating a greater degree of social isolation.

For cognitive ability, this study draws on the multidimensional framework proposed by the Global Aging Research Consensus [36]. A composite cognitive ability score was constructed based on three key dimensions: memory, orientation, and executive. These three dimensions were aggregated to create an overall cognitive ability index, providing a comprehensive assessment of older adults’ functional status across cognitive domains. Notably, although these indicators have been widely adopted in international aging surveys and are supported by solid theoretical and practical foundations [29, 34], variations in specific measurement items and response formats exist across countries. Such differences not only reflect cultural specificity but also highlight the diverse conceptualizations of cognitive ability shaped by distinct social contexts. To improve cross-national comparability, we applied unified coding and standardized all variables to produce scale-free indicators. Nevertheless, caution is warranted when interpreting country-level results of social isolation and cognitive ability, to avoid potential biases arising from cultural and data-related heterogeneity.

#### Covariates

To better explore the relationship between social isolation and cognitive ability, this study includes several time-varying and time-invariant covariates. First, individual demographic characteristics, such as age, gender, education level, marital status, and residence; second, socio-economic characteristics, including pension coverage, health insurance, working status, and income; third, family characteristics such as the number of children. To reduce potential selection bias arising from older adults’ employment decisions based on self-perceived health status, self-rated health was also included as a control variable. Details of the variables and coding strategies are provided in Supplementary Table 1.

#### Country-level indicators

To examine how structural factors at the country level may moderate the relationship between social isolation and cognitive ability in older adults, this study incorporates a range of socioeconomic indicators. These include the Gini index (measuring income inequality within countries), GDP per capita (reflecting economic development), education coverage (capturing access to educational resources), old population density (indicating the degree of population aging), and the poverty rate (representing the proportion of the population living below the poverty line). In addition, two composite indicators of national welfare were included: the World Happiness Index (WHI, representing national-level subjective well-being) and the Human Development Index (HDI, capturing health, education, and living standards). All data were sourced from internationally recognized databases, including the World Bank and the United Nations Development Programme (UNDP), ensuring high levels of cross-national comparability and measurement consistency.

### Statistical analysis

First, linear mixed models (LMM) were separately applied to each country dataset to explicitly account for the hierarchical structure inherent in individual-level data and to mitigate potential omitted variable bias. The LMM approach, which incorporates both random and fixed effects at the individual level, helps to control for individual-specific, time-invariant factors, thus reducing potential bias from unobserved heterogeneity often encountered in traditional regression models [37, 38] Particularly suited for longitudinal analyses, LMM effectively accommodates repeated measurements and simultaneously accounts for both time-varying and time-invariant factors, such as age, gender, socioeconomic status, and educational attainment. Unobservable factors are also indirectly controlled through random effects, thereby enhancing the reliability of the results. Moreover, LMM robustly accounts for time-varying covariates (e.g., income and self-rated health), providing precise estimates by mitigating potential confounding. Compared to traditional OLS regression, LMM exhibits superior adaptability when analyzing time-dependent or hierarchical data structures, capturing the dynamic changes in the dependent variable and the variability in different socio-economic contexts, thereby providing significant statistical support for the in-depth revelation of the multidimensional factors affecting cognitive ability.

Next, data from individual countries were pooled via random effects meta-analysis to estimate an overall effect size and its 95% confidence interval [39]. To further assess the impact of social isolation on cognitive ability, linear mixed-effects models were constructed using social isolation as the key independent variable, with overall cognitive ability and its three subdomains specified as separate dependent variables, allowing for a comprehensive evaluation of their associations. Heterogeneity was assessed using restricted maximum likelihood, which was examined through the I^2^ and H^2^ statistics. I^2^ was utilized as a measure of the percentage of variance in the effect sizes, while H^2^ was employed to ascertain the ratio of the observed variance to the anticipated variance due to sampling error. The presence of I² exceeding 50% or H² greater than 1 is indicative of substantial heterogeneity. To mitigate the impact of discrepancies in sample size on the precision of estimates, the meta-analysis employed a weighted approach, wherein studies with larger sample sizes contributed more significantly to the aggregate effect size [40, 41].

Furthermore, to further strengthen causal inference, this study applies the System Generalized Method of Moments (System GMM) to dynamically model the relationship between social isolation and cognitive ability. This method is particularly well-suited for unbalanced panel data with short time spans and large individual dimensions [7]. By leveraging lagged values of endogenous regressors as instruments, System GMM effectively addresses potential endogeneity arising from reverse causality, omitted variable bias, and measurement error [42, 43]. Given the likely bidirectional relationship between social isolation and cognition, and the influence of unobserved individual traits, GMM provides a robust framework for identifying dynamic causal effects. To ensure model robustness and identification validity, several diagnostic tests were conducted, including comparisons of first- and second-order lag structures, alternative specifications of instrumental variables using lagged differences and levels, and robustness checks by replacing the dependent variable with different cognitive subdomains. The models passed the Hansen test for over-identification and showed no evidence of second-order serial correlation (AR(2)), confirming instrument validity and correct model specification. Sensitivity analyses revealed that despite slight variations in model specification, the direction and significance of the main coefficients remained consistent, reinforcing the robustness of the findings. Due to GMM’s requirement for consecutive observations, only individuals with valid data for at least two waves were retained. We addressed missing data via listwise deletion and excluded countries with major data discontinuities (e.g., Estonia, Hungary), resulting in a final sample of 17 countries and 47,483 person-wave observations, representing 46.7% of the total sample. Although the GMM subsample is smaller than that used in the linear mixed-effects models, it retains a representative distribution in terms of both country coverage and individual characteristics, with no observable selection bias. Moreover, a comparison of GMM results with those from LMM and OLS models yielded highly consistent estimates in both direction and significance, providing further validation for the study’s conclusions. All analyses were conducted using Stata v.17.

Further analyses were conducted at macro and micro levels to explore moderating factors. At the macro level, meta-regression using country-level variables explored sources of heterogeneity [38, 44]. Multilevel modeling was also employed across integrated datasets to explore national-level mechanisms. At the micro level, interaction terms within LMM examined moderating roles of individual demographic and socioeconomic characteristics [45–47].

Finally, multiple sensitivity analyses were performed to assess the robustness of the findings. Specifically: (1) Fixed-effects models were additionally performed to confirm the robustness of the main findings; (2) To address potential biases arising from missing data, multiple imputation was performed for key variables, including social isolation, cognitive ability, and major time-varying covariates. To account for cross-national cultural and institutional heterogeneity, imputations were conducted separately within each national cohort, thereby enhancing the internal consistency of the imputed values. The imputation model incorporated mortality status, functional impairment, and baseline cognitive scores to adequately account for health-related attrition that might affect sample retention. The main analyses were subsequently re-estimated using the multiply imputed datasets; (3) To maintain dataset comparability, the primary analyses included respondents aged ≥ 60 years. However, relatively younger respondents (aged 60–64 years) might influence results; thus, analyses were further restricted to respondents aged ≥ 65 years to verify the robustness of findings using the 24-country cohort data.

## Results

### Participants

Supplementary Table 2 summarizes the characteristics of the final analytical sample (24 countries, *N* = 101,581) utilized for the examination of the baseline association between social isolation and cognitive ability. Baseline characteristics demonstrated considerable cross-national heterogeneity. The mean age of participant in each country ranged from 64.9 years (China) to 73.9 years (USA), and women accounted for more than half of participants in all countries except China. Educational attainment displayed a clear gradient across countries by economic development, with higher education levels among older adults in developed countries compared to developing countries. Labor force participation varied significantly, from 7.9% in Slovenia to 58.2% in China, reflecting generally low employment rates among older adults. Social isolation scores among individuals aged ≥ 60 years also exhibited substantial cross-national disparities, with baseline mean scores ranging from 0.8 in China and the Netherlands to 1.8 in Korea. Regarding cognitive ability, most countries showed generally above-average cognitive performance, except for China and Portugal. To minimize potential confounding, linear mixed models adjusted for multiple covariates were estimated separately for each country. Subsequently, a meta-analysis synthesizing estimates from these linear mixed models across the five longitudinal aging cohorts was performed to systematically assess the association between social isolation and cognitive ability among older adults. 

### Meta-analysis results of social isolation and cognitive ability

**Fig. 1:**
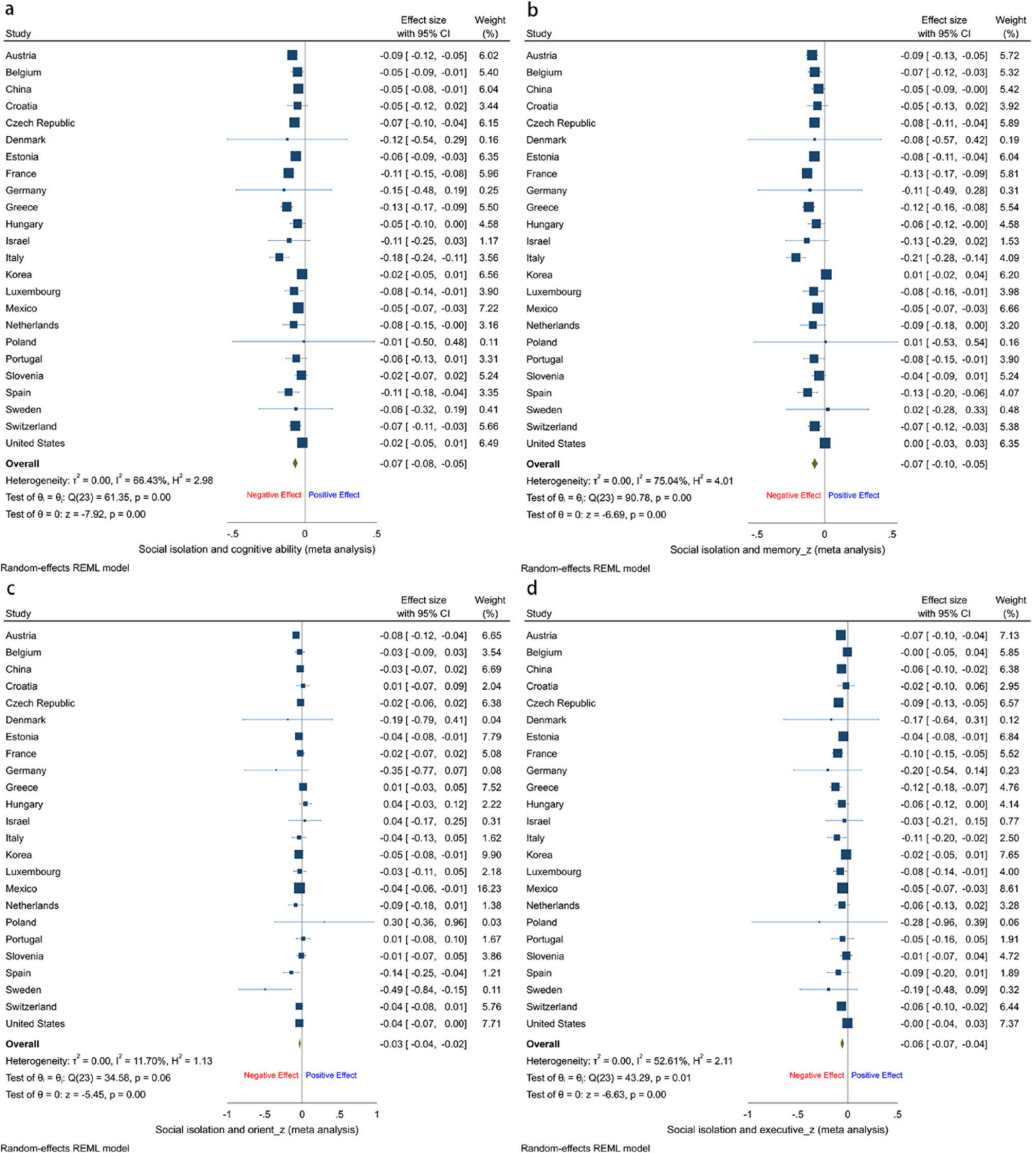
Results of a meta-analysis of social isolation and cognitive ability in older adults. **A**, Cognitive ability. **B**, Memory ability. **C**, Orientation ability. **D**, Executive ability.

As illustrated in Fig. 1, social isolation was significantly negatively associated with overall cognitive ability (pooled coefficient = −0.07; 95% CI = −0.08, −0.05; I² = 66.43%; H² = 2.98). The I² and H² values suggest that the variability in the effect sizes is primarily attributable to heterogeneity across countries, rather than from sampling error alone. Further analysis by cognitive domain confirmed consistent and statistically significant negative associations between social isolation and memory (pooled coefficient = −0.07; 95% CI = −0.10, −0.05; I² = 75.04%; H² = 4.01), orientation (pooled coefficient = −0.03; 95% CI = −0.04, −0.02; I² = 11.70%; H² = 1.13), and executive (pooled coefficient = −0.06; 95% CI = −0.07, −0.04; I² = 52.61%; H² = 2.11). Overall, the meta-analytic findings indicate a consistent negative impact of social isolation on cognitive ability across multiple domains, with substantial cross-national variability.

### Dynamic association between social isolation and cognitive ability: endogeneity correction via system GMM

Although linear mixed models initially indicated associations between social isolation and cognitive ability among older age groups, the validity of their causal inference is still constrained by potential endogeneity issues. To address biases arising from omitted variables and reverse causality, this study adopted a dynamic panel modeling approach using System GMM. Specifically, lagged cognitive ability measures (first-order lags, AR(1)) were incorporated as instrumental variables, effectively reducing endogeneity bias in parameter estimation [42]. Based on the panel survey structure, the final analytical sample includes 17 countries with complete data from at least two waves (*n* = 47,483).

**Fig. 2:**
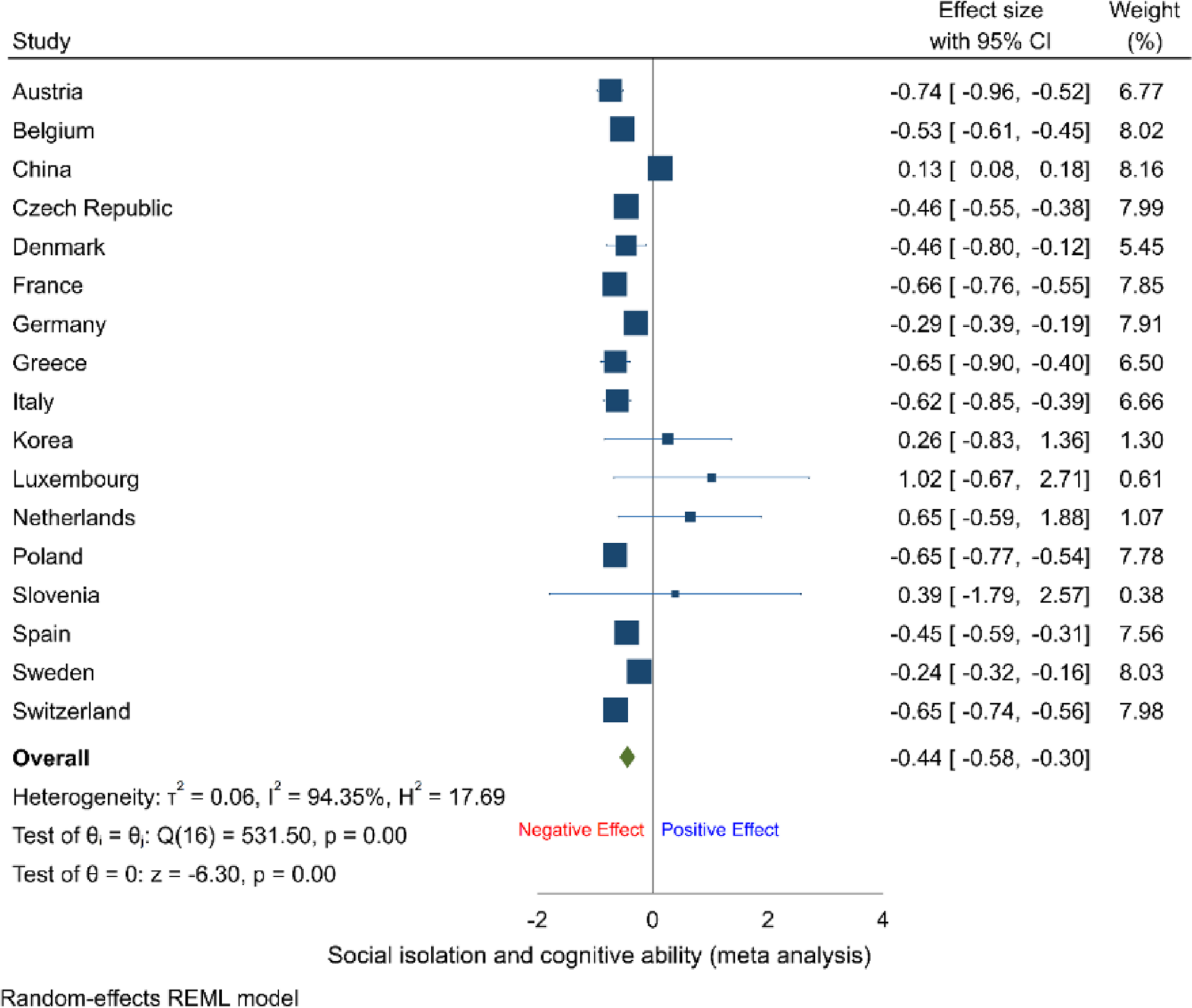
Results of GMM analyses of social isolation and cognitive ability

The GMM estimates depicted in Fig. 2 demonstrate that, after controlling for individual-level fixed effects and temporal trends, the negative association between social isolation and cognitive ability remained statistically significant and substantial (pooled coefficient = −0.44; 95% CI = −0.58, −0.30; I² = 94.35%; H² = 17.69), providing supporting evidence for the robustness of this association. Moreover, the Hansen J tests yielded p-values greater than 0.1, providing strong evidence that the instrumental variables did not exhibit overidentification concerns. The AR(2) test further indicated no second-order serial correlation among residuals, verifying that the chosen instrument satisfied both exclusion and relevance conditions. These diagnostic results support the statistical adequacy and validity of the dynamic model specification.

### Country-level factors

In the preceding meta-analysis, observed heterogeneity in effects across countries may be attributable to variations in socioeconomic structures, cultural contexts, and stages of population aging. Further analyses revealed that country-level factors are closely related to the association between social isolation and cognitive ability, particularly socioeconomic variables such as the Gini index, GDP per capita, education coverage, old population density, and poverty rate, as well as social welfare variables including the WHI and the HDI. Specifically, country-level social isolation indices exhibited a weak positive correlation with the poverty rate (*r* = 0.05), and significant negative correlations with the Gini index (*r* = −0.32), WHI (*r* = −0.43), GDP per capita (*r* = −0.44), education coverage (*r* = −0.02), old population density (*r* = −0.18), and HDI (*r* = −0.25) (see Supplementary Fig.2).

To further explore whether these country-level factors moderate the observed association between social isolation and cognitive ability among older adults, this study integrated multiple national datasets and performed analyses using multilevel modeling. After adjusting for potential confounders, the results indicate that country-level differences have a substantial impact on the ability to explain the overall variation, with an overall explanation rate of 19.7%.

On this basis, these country-level variables were included as predictors in meta-regression analyses to assess their potential impact on effect heterogeneity (see Extended Data Figs. S3, S4, S5 and S6). Results showed no significant associations between effect size and the Gini index, GDP per capita, education coverage, HDI, or poverty rate. Additionally, with the exception of orientation, effect sizes across cognitive domains were not significantly associated with WHI. However, old population density exhibited a significant negative correlation with the magnitude of the association between social isolation and cognitive ability: each unit increase in old population density was associated with a 0.02-point reduction in the strength of this relationship.

**Fig. 3:**
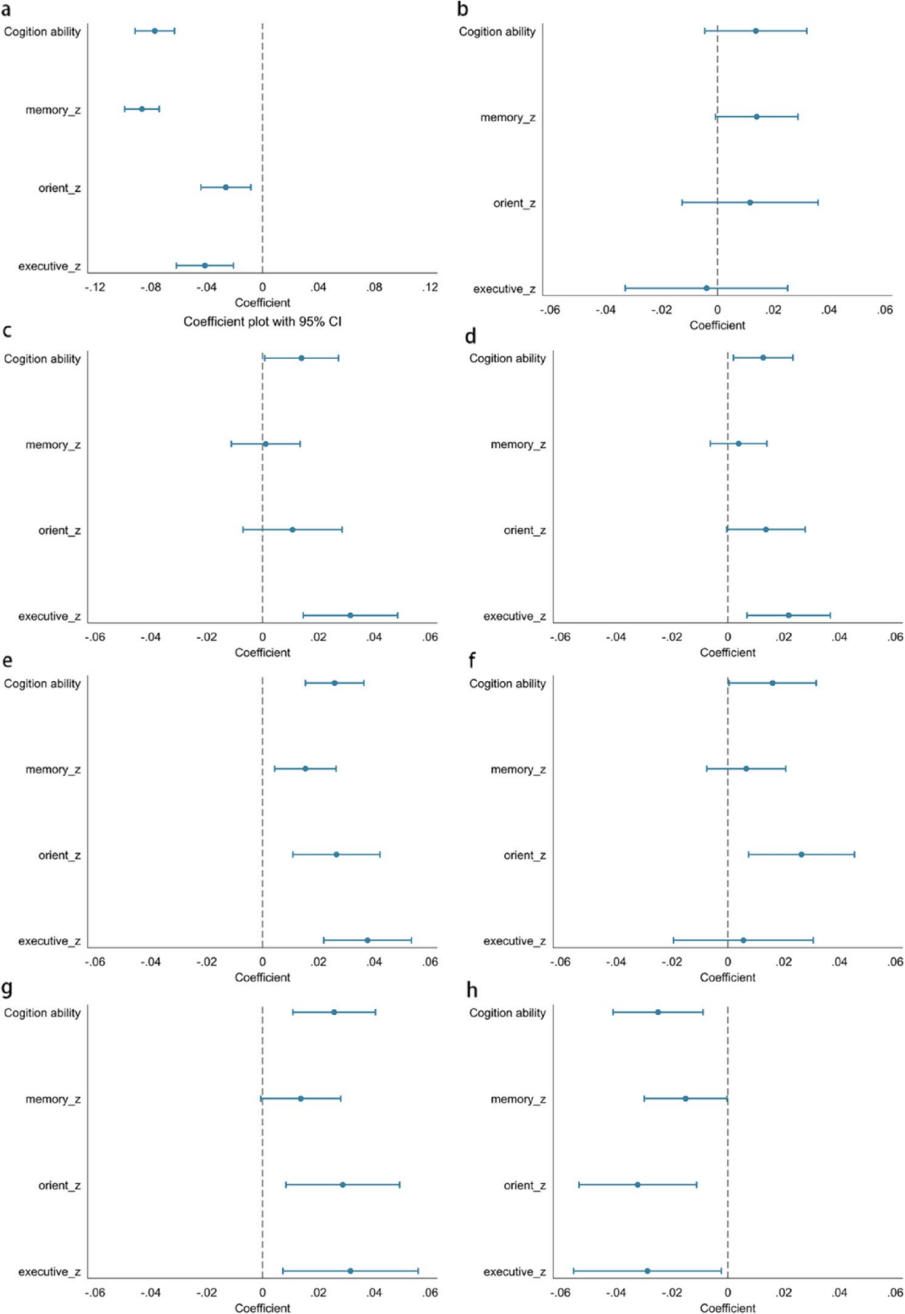
Results of the analysis of the moderating effects of country-level factors. Data are presented as coefficients and 95% confidence intervals. **A**, No interaction. **B**, Interacting with Gini index. **C**, Interacting with WHI. **D,** Interacting with GDP per capita. **E**, Interacting with education coverage. **F**, Interacting with old population density. **G**, Interacting with HDI. **H**, Interacting with poverty rate.

Finally, multilevel analyses further examined whether country-level factors moderated the association between social isolation and cognitive ability among older adults. Results presented in Fig. 3 indicated positive moderating effects of the WHI, GDP per capita, education coverage, old population density, and HDI, whereas the poverty rate exhibited a negative moderating effect (β = −0.03; 95% CI = −0.04, −0.01). These findings suggest that although the moderating effects of country-level factors are relatively modest, they nevertheless exert a meaningful influence on the relationship between social isolation and cognitive ability among older adults.

### Subgroup analysis

**Fig. 4:**
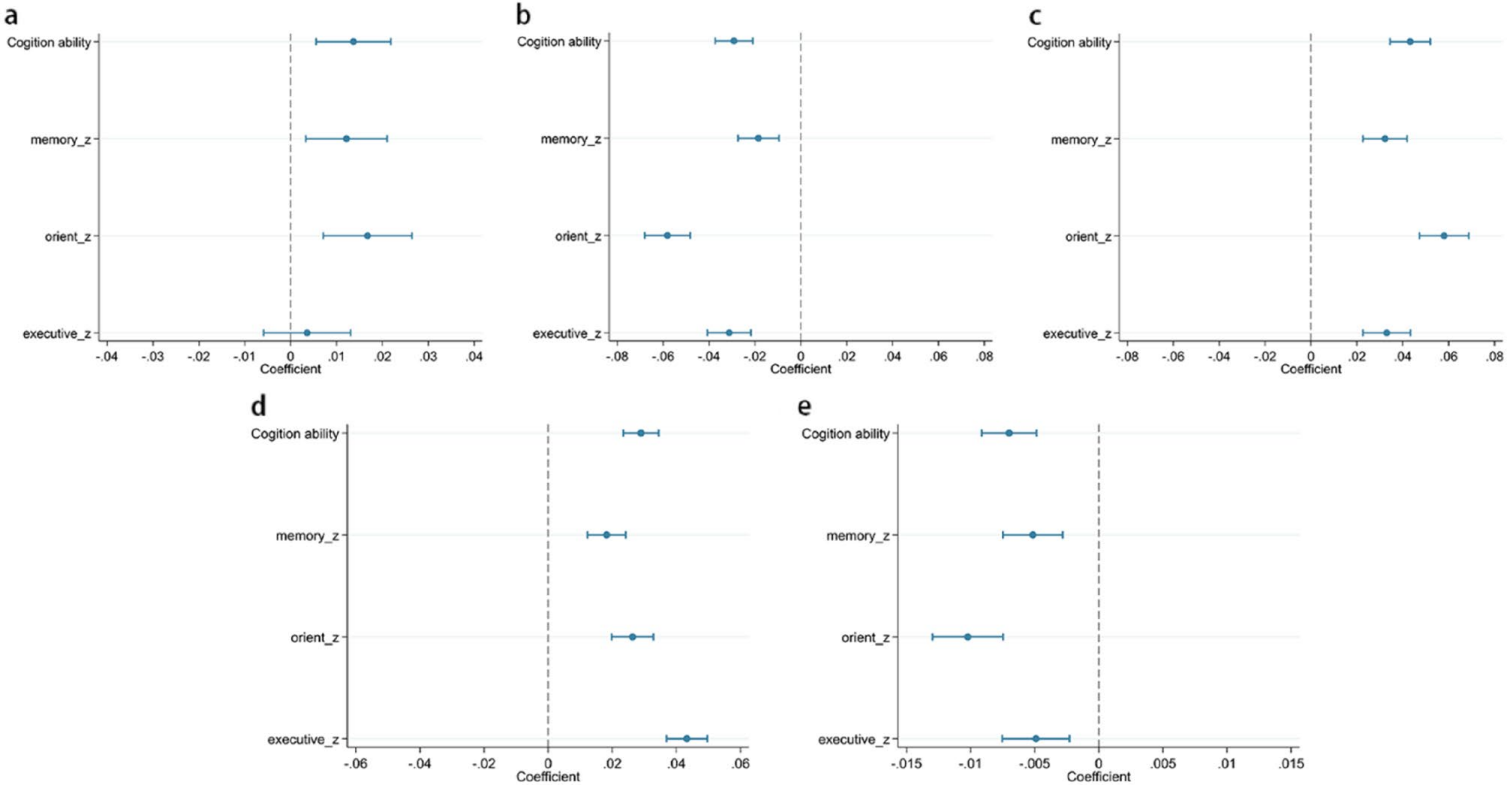
Results of subgroup analyses. Data are presented as coefficients and 95% confidence intervals. **A**, Interacting with Gender. **B**, Interacting with Age. **C**, Interacting with Working status. **D**, Interacting with Education. **E**, Interacting with Children.

This study systematically examined the moderating role of individual-level factors in the association between social isolation and cognitive ability through interaction analyses, as illustrated in Fig. 4. With respect to gender differences, female older adults exhibited greater vulnerability to the negative cognitive effects of social isolation compared to older men, particularly in memory and orientation domains (overall cognition: β = 0.01, 95% CI = 0.01, 0.02; memory: β = 0.01, 95% CI = 0.00, 0.02; orientation: β = 0.02, 95% CI = 0.01, 0.03). However, no significant gender difference was found for executive ability. Regarding age groups, individuals aged 75 years and above demonstrated a more pronounced cognitive deterioration associated with social isolation, with significantly stronger negative associations across all cognitive domains (overall cognition: β = −0.03, 95% CI = −0.04, −0.02; memory: β = −0.02, 95% CI = −0.03, −0.01; orientation: β = −0.06, 95% CI = −0.07, −0.05; executive: β = −0.03, 95% CI = −0.04, −0.02).

In terms of socioeconomic status, older adults who were unemployed or economically inactive (overall cognition: β = 0.04, 95% CI = 0.03, 0.05; memory: β = 0.03, 95% CI = 0.02, 0.04; orientation: β = 0.06, 95% CI = 0.05, 0.07; executive: β = 0.03, 95% CI = 0.02, 0.04) and those with lower educational attainment (overall cognition: β = 0.03, 95% CI = 0.02, 0.03; memory: β = 0.02, 95% CI = 0.01, 0.02; orientation: β = 0.03, 95% CI = 0.02, 0.03; executive: β = 0.04, 95% CI = 0.04, 0.05) exhibited significantly exacerbated negative associations between social isolation and cognitive performance across all examined domains. Finally, older adults with a greater number of children also showed significantly stronger negative associations between social isolation and cognitive outcomes (overall cognition: β = −0.01, 95% CI = −0.01, −0.00; memory: β = −0.01, 95% CI = −0.01, −0.00; orientation: β = −0.01, 95% CI = −0.01, −0.01; executive: β ≈ 0.00, 95% CI = −0.01, −0.00).

### Sensitivity analysis

Multiple sensitivity analyses were conducted to assess the robustness of the study’s findings. Initially, to further evaluate the stability of findings under alternative modeling assumptions, fixed-effects models were employed as a robustness check, revealing regression coefficients strongly consistent with the baseline results (Extended Fig. 7). Second, multiple imputation was applied to address missing data, and the imputed results remained consistent with those based on the original dataset (see Extended Fig. 8), further supporting the validity of the model estimates. Finally, to examine whether the results were sensitive to variations in defining the old population, the minimum age criterion for inclusion was increased to 65 years, and the meta-analysis was replicated (Extended Fig. 9). The negative impact of social isolation on cognitive ability remained statistically significant under this revised criterion. Collectively, these sensitivity analyses confirm that the main findings are robust across various model specifications, missing data treatments, and age thresholds, supporting their generalizability.

## Discussion

Drawing on longitudinal data from five representative aging cohorts across 24 countries, this study systematically investigated the dynamic associations between social isolation and cognitive ability in older adults. Methodologically, it employs rigorous strategies to ensure robust causal identification; theoretically, it explores the tension between the universality of effects and their contextual variation. The results consistently demonstrate a significant negative association between social isolation and cognitive ability across multiple model specifications and sensitivity analyses. Importantly, the study moves beyond establishing main effects to highlight the conditional nature of this relationship: the cognitive consequences of social isolation differ across national institutional structures, cultural norms, and social support systems. We thus contend that the causal pathway linking social isolation to cognitive ability is inherently context-dependent. This perspective not only advances theoretical debates on cross-cultural generalizability but also informs the development of more context-sensitive and targeted policy interventions [2].

From a theoretical viewpoint, the mechanisms underlying these negative associations offer context-specific empirical support for both Ecological Systems Theory and Social Embeddedness Theory. Ecological systems theory posits that cognitive development is shaped by the dynamic interplay among multiple layers of the social environment in which individuals are embedded, including the microsystem (e.g., family interactions), mesosystem (e.g., community and neighborhood ties), and exosystem (e.g., institutional and policy structures) [27]. Building on this framework, our findings reveal that, at the micro- and mesosystem levels, social isolation reduces the frequency and quality of older adults’ interactions with family and neighborhood networks, thereby limiting cognitive stimulation and depleting cognitive reserve [48, 49]. At the exosystem level, cross-national differences in historically embedded institutional arrangements and social policies shape disparities in access to social support and mental health services, which in turn influence cognitive vulnerability in later life [50]. From the perspective of Social Embeddedness Theory, access to cognitive resources, emotional support, and informational feedback through social networks plays a vital role in sustaining cognitive ability [51, 52]. When older adults experience weakened, fragmented, or disconnected social ties, their access to these resources diminishes, thereby reducing external stimulation and emotional regulation, further exacerbating cognitive decline. Furthermore, by integrating a structural perspective to illustrate how multidimensional deficits in social interaction resources collectively influence cognitive decline trajectories among older adults, this study extends and deepens the application and explanatory scope of Ecological Systems Theory and Social Embeddedness Theory in the cognitive health field. It highlights that the lack of social resources does not affect cognitive ability in a single, compartmentalized manner. Rather, it triggers the weakening of cognitive resources through the systematic disintegration of the social network structure, thereby accelerating the cognitive aging process.

Another critical innovation of this study is its systematic exploration of cross-national differences in the association between social isolation and cognitive ability. Notably, although the Chinese sample had the youngest average age (64.9 years), their baseline cognitive performance was markedly lower than that of other countries—particularly the United States, where the average age was 73.9 years. This finding runs counter to the conventional expectation that younger individuals exhibit higher cognitive ability. Such a counterintuitive pattern may stem from a combination of factors, including lower overall educational attainment among older adults in China, cultural differences in the applicability of cognitive assessments, deep-rooted historical and cultural influences, and varying levels of socioeconomic development [53, 54]. Consistent with this observation, System GMM analysis also revealed country-specific variations. For instance, a counterintuitive positive association between social isolation and cognitive function emerged in the Chinese sample. This may reflect the enduring strength of family-based cognitive support embedded in the mesosystem, characteristic of Chinese culture’s tightly knit kinship networks. In contrast, in countries with comprehensive welfare systems such as Luxembourg and the Netherlands, institutionalized community services and policy infrastructures at the exosystem level appear to offset the cognitive risks associated with reduced familial or neighborhood interaction [55, 56]. These divergences can be further interpreted through the lens of historical institutionalism: differences in welfare regime trajectories, the timing and scope of institutional expansion, and the historical accumulation of social capital have led to structurally unequal distributions of cognitive support across nations [57, 58]. For example, Nordic countries established extensive eldercare systems and localized support mechanisms early on, whereas China’s welfare transition involved a process of de-institutionalization and re-familialization, increasing older adults’ reliance on informal care and kinship-based networks [17, 59]. Taken together, these findings underscore the context-dependent nature of social isolation’s impact on cognition. They highlight the layered influence of national histories, institutional legacies, and cultural norms. Thus, cross-national analyses must be grounded in contextual understandings of country-specific social structures, value systems, and policy environments, and should account for the heterogeneity in how social isolation operates across different societies.

Further analysis suggests that country-level factors, including economic development, income inequality, old population density, and social welfare systems, significantly moderated the relationship between social isolation and cognitive outcomes. Meta-regression analysis further revealed a negative association between population aging and the cognitive impact of social isolation: countries with higher old population densities exhibited weaker negative associations. This may be due to the fact that countries with higher levels of aging have established relevant social policies (e.g., age-friendly city programs) earlier in the construction of an aging society, providing more extensive channels and resources for social participation, thus partially mitigating cognitive risks associated with social isolation [60, 61]. National-level macro-structural factors moderate this relationship because they shape the broader social and institutional context in which individuals are embedded, systematically affecting their access to social resources. For instance, higher levels of economic development or lower income inequality are often associated with more robust healthcare systems, broader educational access, and richer opportunities for social engagement—all of which enhance individuals’ capacity to cope with the risks posed by social isolation. Culturally, welfare systems and mechanisms of social integration not only provide formal support and accessible services but also influence cultural norms and social expectations, thereby shaping how individuals interpret and respond to isolation and its cognitive consequences. Building on this perspective, our study extends the applicability of Ecological Systems Theory by showing that cognitive health is shaped not only by micro-level (e.g., family, neighborhood) and meso-level (e.g., community networks) factors, but also by macro-level institutions and cultural norms. While the moderating effects of certain national-level variables may be modest and our analysis remain exploratory, the findings underscore the critical role of macro-structural conditions in explaining cognitive outcomes and offer valuable insights for future cross-cultural research on aging.

In addition to cross-national heterogeneity, this study systematically revealed subgroup differences at the individual level in the relationship. It can be found that cognitive impairment induced by social isolation was particularly prominent among socially disadvantaged subgroups, including older adults with lower socioeconomic status, non-working individuals, older age groups, and women, aligning closely with cognitive reserve and cumulative disadvantage theories. Specifically, non-working or less-educated older adults typically possess fewer cognitive reserves and resources, making it difficult for them to obtain sufficient alternative cognitive resources and social support in the face of social isolation. Consequently, these individuals experience notably exacerbated cognitive decline [62]. Additionally, advanced-age individuals, experiencing physiological and adaptive declines, are more vulnerable to the emotional stress and scarcity of social resources triggered by social isolation, accelerating cognitive deterioration. Significant gender differences were also observed: older women exhibited greater cognitive impairment, especially in memory and orientation, under social isolation. This phenomenon may pertain to the fact that women tend to have longer life expectancies, higher rates of solo living, and a greater probability of encountering diminished social interaction networks, a paucity of social support resources, and an increased propensity for mental health challenges as they transition into their senior years [63, 64]. Another novel finding of this study is that older adults with more children experienced greater cognitive decline under social isolation. This finding questions the conventional assumption that having more children necessarily offers protective benefits through family support. In fact, a larger number of children may raise older adults’ expectations for support. When actual support is lacking—due to children’s busy schedules, geographic distance, or weakened intergenerational ties—Such unmet expectations may heighten feelings of loneliness and emotional stress, which could potentially contribute to worse cognitive outcomes [65, 66]. From a social-psychological perspective, the quality of family relationships, fairness in caregiving, and frequency of communication often matter more than the number of children. Without emotional closeness or meaningful interaction, even those with many children may remain functionally isolated despite being structurally embedded. Moreover, having more children can introduce conflicts and caregiving imbalances, further destabilizing emotional and cognitive well-being [67, 68]. These findings highlight that in promoting cognitive health and social integration, the number of children should not be treated as a simple indicator of family support. Instead, attention should be directed toward the quality of intergenerational relationships, emotional connection, and interaction frequency to better buffer the negative effects of social isolation.

A key strength of this study lies in utilizing large-scale longitudinal aging data from 24 countries, effectively addressing limitations inherent in single-country analyses. This approach allowed systematic identification of the cross-national impact of social isolation on cognition and exploration of how country-level factors moderate this relationship. Furthermore, the combined use of linear mixed models, multinational meta-analysis, and System GMM estimation enhanced the robustness and comparability of results while reducing endogeneity concerns, thereby offering more credible evidence for longitudinal associations. Additionally, extensive subgroup analyses illuminated how diverse individual and socioeconomic contexts moderate these relationships, advancing theoretical understanding and providing empirical evidence to inform targeted interventions and global healthy aging policies.

Nevertheless, this study has several limitations. First, although strategies such as System GMM were employed to mitigate reverse causality and omitted variable bias, the use of observational data and a non-experimental design inherently limits the precise identification of causal relationships. The possibility of residual confounding cannot be fully excluded. Future research could strengthen causal inference through randomized controlled trials or natural experiments. Second, given data constraints, the sample primarily includes middle- and high-income countries, which may limit the generalizability of the findings. The limited variation in national-level factors, such as economic development and welfare systems, also constrains the scope of moderation analysis. Expanding research to include low-income countries and incorporating more context-sensitive macro indicators would enhance the cross-national relevance and explanatory power of future studies. Third, despite efforts to standardize measurements across cultures, differences in cultural understandings of social isolation and cognitive ability may introduce biases. Future research should enhance cross-cultural adaptability of measurement instruments. Fourth, genetic factors, early-life experiences, and psychological characteristics were not included due to database limitations, potentially constraining the comprehensiveness of the conclusions. Fourth, variability across countries in the frequency and intervals of follow-up assessments may compromise the consistency in capturing temporal dynamics such as social isolation. Future studies should aim for greater consistency in cross-national data collection methods. Lastly, the meta-analysis revealed substantial cross-national heterogeneity (I² >50%). Although random-effects models were employed to accommodate this variability, pooled estimates may not accurately reflect country-specific contexts. Future research should further integrate country-level characteristics using stratified analyses or multilevel modeling to better identify and explain heterogeneity.

## Conclusion and policy implications

In conclusion, this study, leveraging cross-national longitudinal data and advanced econometric methods, provides robust evidence for a broadly applicable negative association between social isolation and cognitive ability among older adults. Given global population aging trends, policymakers may consider prioritizing multi-level social integration strategies, enhancing opportunities for social engagement and network building, particularly in nations with lower economic development and underdeveloped welfare systems. Special attention should be given to socially disadvantaged older populations, leveraging community interventions, strengthened social protections, and technological innovations to create inclusive community environments, fostering healthy aging and contributing to sustainable development goals.

## Supplementary Information

Supplementary Material 1.

## Data Availability

The original datasets used in this study can be accessed through the following websites: CHARLS (https://charls.charlsdata.com/pages/data/111/zh-cn.html), KLoSA (https://survey.keis.or.kr/eng/klosa/klosa01.jsp), MHAS (https://www.mhasweb.org/), SHARE (https://share-eric.eu/data/data-access. Html), HRS (https://hrs.isr.umich.edu/data-products).

## Abbreviations

GMM: Generalized method of moments
WHO: World health organization
LMM: Linear mixed models
AR: Autoregressive model
GDP: Gross domestic product
WHI: World happiness index
HDI: Human development index
UNDP: United nations development programme
