# Supplementary material for: Social isolation and cognitive decline in older adults: a longitudinal study across 24 countries
Source: BMC Geriatr. 2025 Oct 14;25:775. doi: 10.1186/s12877-025-06430-6 (PMC12522220; doi:10.1186/s12877-025-06430-6)
Supplement: Supplementary file 1 — Supplementary Material 1. [file 12877_2025_6430_MOESM1_ESM.docx]

**Supplementary Table 1** Variable definition and assignment

| Variable name | Variable definition and assignment |
| --- | --- |
| Dependent variable: Cognitive ability | |
| Memory ability | Situational memory ability, including immediate memory test and delayed memory test, summing up the test results; higher scores indicate better memory ability. |
| Orientation ability | Orientation ability test, including time and place orientation questions, summing up the results; higher scores indicate better time and space perception ability. |
| Executive ability | Attention and numeracy, including numerical breadth and semantic fluency tests, with results summed; higher scores indicate better executive ability. |
| Core explanatory variable | |
| Social isolation | Values were assigned based on the scale regarding social isolation, including marital status, residence pattern, family interaction, and social participation, with higher scores indicating greater social isolation. |
| Covariates | |
| Age | Age of respondent at time of interview (years). |
| Gender | Gender of respondent; values: female = 0, male = 1. |
| Education | Respondent's level of education; values: less than lower secondary = 1, upper secondary and vocational = 2, tertiary = 3. |
| Marital status | Current marital or cohabitation status of the respondent; values: not living with partner = 0, living with partner = 1. |
| Region | Type of area where respondent lives; values: urban = 0, rural = 1. |
| Pension | Whether the respondent participates in an old-age insurance or state pension scheme; values assigned: not participating = 0, participating = 1. |
| Insurance | Whether the respondent has health insurance or private health insurance; value assigned: not participating = 0, participating = 1. |
| Working status | Whether the respondent is currently employed; assignment: no = 0, yes = 1. |
| Income status | The respondent's current income (logarithmic). |
| Children | Number of living children of the respondent (number). |
| Self-reported health | The respondent's subjective assessment of their own health; the higher the value, the better the self-rated health. |

**Supplementary Table 2** Basic demographics by country in percentages (%) or mean (s.d.)

| Country | Social isolation  (s.d.) | | Gender  (%) | | Age  (s.d.) | | Education  (%) | | | Working status  (%) | | Cognitive ability  (s.d.) | | N |
| --- | --- | --- | --- | --- | --- | --- | --- | --- | --- | --- | --- | --- | --- | --- |
|  | Mean  (s.d.) | Range | Female | Male | Mean  (s.d.) | Range | Low | Middle | High | Working | Not  working | Mean  (s.d.) | Range |  |
| Austria | 1.2  (1.08) | 0-4 | 58.1 | 41.9 | 69.5  (7.82) | 60-96 | 25.2 | 49.1 | 25.7 | 13.8 | 86.2 | 18.2  (4.40) | 0-29 | 4112 |
| Belgium | 1.2  (1.06) | 0-4 | 53.6 | 46.4 | 69.8  (8.43) | 60-97 | 42.0 | 25.6 | 32.3 | 18.3 | 81.7 | 17.3  (4.47) | 0-29 | 5024 |
| China | 0.8  (0.79) | 0-4 | 44.7 | 55.3 | 64.9  (5.42) | 60-101 | 88.7 | 9.2 | 2.1 | 58.2 | 41.8 | 13.8  (4.43) | 0-28 | 9078 |
| Croatia | 1.3  (0.89) | 0-4 | 53.1 | 46.9 | 69.0  (7.31) | 60-97 | 57.9 | 27.4 | 17.7 | 10.8 | 89.2 | 16.4  (4.39) | 0-29 | 2367 |
| Czech  Republic | 1.3  (1.05) | 0-4 | 58.2 | 41.8 | 68.8  (7.39) | 60-100 | 38.6 | 47.8 | 13.6 | 21.4 | 78.6 | 17.3  (4.05) | 0-28 | 5555 |
| Denmark | 0.9  (1.04) | 0-4 | 52.2 | 47.8 | 69.0  (8.00) | 60-100 | 19.0 | 39.8 | 41.2 | 37.5 | 62.5 | 18.3  (4.03) | 0-29 | 3081 |
| Estonia | 1.5  (1.02) | 0-4 | 60.5 | 39.5 | 69.3  (7.59) | 60-100 | 31.6 | 47.2 | 21.2 | 29.0 | 71.0 | 16.7  (4.51) | 0-29 | 5692 |
| France | 1.3  (1.10) | 0-4 | 55.9 | 44.1 | 69.9  (8.51) | 60-98 | 46.5 | 32.7 | 20.8 | 16.5 | 83.5 | 16.6  (4.52) | 0-28 | 4480 |
| Germany | 1.1  (1.04) | 0-4 | 50.6 | 49.4 | 69.1  (7.60) | 60-99 | 13.0 | 55.4 | 31.6 | 30.9 | 69.1 | 17.8  (4.07) | 0-29 | 4647 |
| Greece | 1.3  (0.92) | 0-4 | 53.5 | 46.5 | 69.8  (7.90) | 60-94 | 56.0 | 26.0 | 18.0 | 20.5 | 79.5 | 16.3  (3.88) | 0-29 | 3824 |
| Hungary | 1.4  (0.98) | 0-4 | 55.6 | 44.4 | 68.8  (7.20) | 60-99 | 32.7 | 51.0 | 16.3 | 11.7 | 88.3 | 16.4  (4.30) | 0-29 | 2369 |
| Israel | 1.0  (0.95) | 0-4 | 55.1 | 44.9 | 71.3  (8.25) | 60-105 | 32.3 | 30.1 | 37.6 | 32.3 | 67.7 | 16.8  (4.61) | 0-29 | 2070 |
| Italy | 1.2  (0.88) | 0-4 | 52.4 | 47.6 | 69.7  (7.56) | 60-102 | 72.5 | 19.9 | 7.5 | 14.8 | 85.2 | 15.5  (4.43) | 0-28 | 4684 |
| Korea | 1.8  (0.84) | 0-4 | 57.2 | 42.8 | 68.8  (7.50) | 60-103 | 70.0 | 22.4 | 7.6 | 37.0 | 63.0 | 17.2  (3.94) | 0-21 | 5778 |
| Luxembourg | 1.0  (0.99) | 0-4 | 51.5 | 48.5 | 68.4  (7.76) | 60-96 | 46.7 | 37.5 | 15.8 | 13.0 | 87.0 | 17.5  (4.47) | 0-29 | 1469 |
| Mexico | 1.5  (0.98) | 0-4 | 55.6 | 44.4 | 68.4  (6.98) | 60-98 | 89.2 | 2.5 | 8.3 | 31.6 | 68.4 | 14.2  (4.15) | 0-27 | 8957 |
| Netherlands | 0.8  (0.99) | 0-4 | 52.8 | 47.2 | 70.1  (7.59) | 60-99 | 46.2 | 24.8 | 29.0 | 25.5 | 74.5 | 17.7  (3.94) | 2-29 | 2671 |
| Poland | 1.3  (0.88) | 0-4 | 54.6 | 45.4 | 69.4  (7.81) | 60-97 | 31.8 | 57.2 | 11.0 | 16.4 | 83.6 | 15.4  (4.36) | 0-29 | 2844 |
| Portugal | 1.1  (0.85) | 0-4 | 51.2 | 48.8 | 68.5  (7.21) | 60-95 | 80.2 | 9.6 | 10.2 | 14.9 | 85.1 | 14.4  (4.43) | 0-29 | 1423 |
| Slovenia | 1.0  (0.96) | 0-4 | 54.8 | 45.2 | 69.0  (7.82) | 60-99 | 34.6 | 49.2 | 16.2 | 7.9 | 92.1 | 16.1  (4.26) | 0-29 | 4251 |
| Spain | 1.1  (0.83) | 0-4 | 50.5 | 49.5 | 70.4  (8.35) | 60-102 | 79.8 | 9.2 | 11.0 | 17.6 | 82.4 | 14.1  (4.61) | 0-27 | 4818 |
| Sweden | 1.0  (1.01) | 0-4 | 52.4 | 47.6 | 70.5  (7.88) | 60-102 | 38.2 | 31.9 | 29.9 | 36.8 | 63.2 | 17.7  (4.01) | 0-29 | 4100 |
| Switzerland | 1.0  (1.06) | 0-4 | 52.7 | 47.3 | 69.1  (8.00) | 60-101 | 21.1 | 62.4 | 16.5 | 39.8 | 60.2 | 18.7  (4.00) | 1-29 | 3000 |
| USA | 1.0  (1.06) | 0-4 | 59.3 | 40.7 | 73.9  (6.22) | 60-101 | 15.6 | 61.6 | 22.8 | 21.4 | 78.6 | 18.9  (3.97) | 3-31 | 5287 |

**Note**: The table shows baseline demographics where baseline indicates the first wave at which each participant completed the survey and therefore does not relate to a single year of data.

**
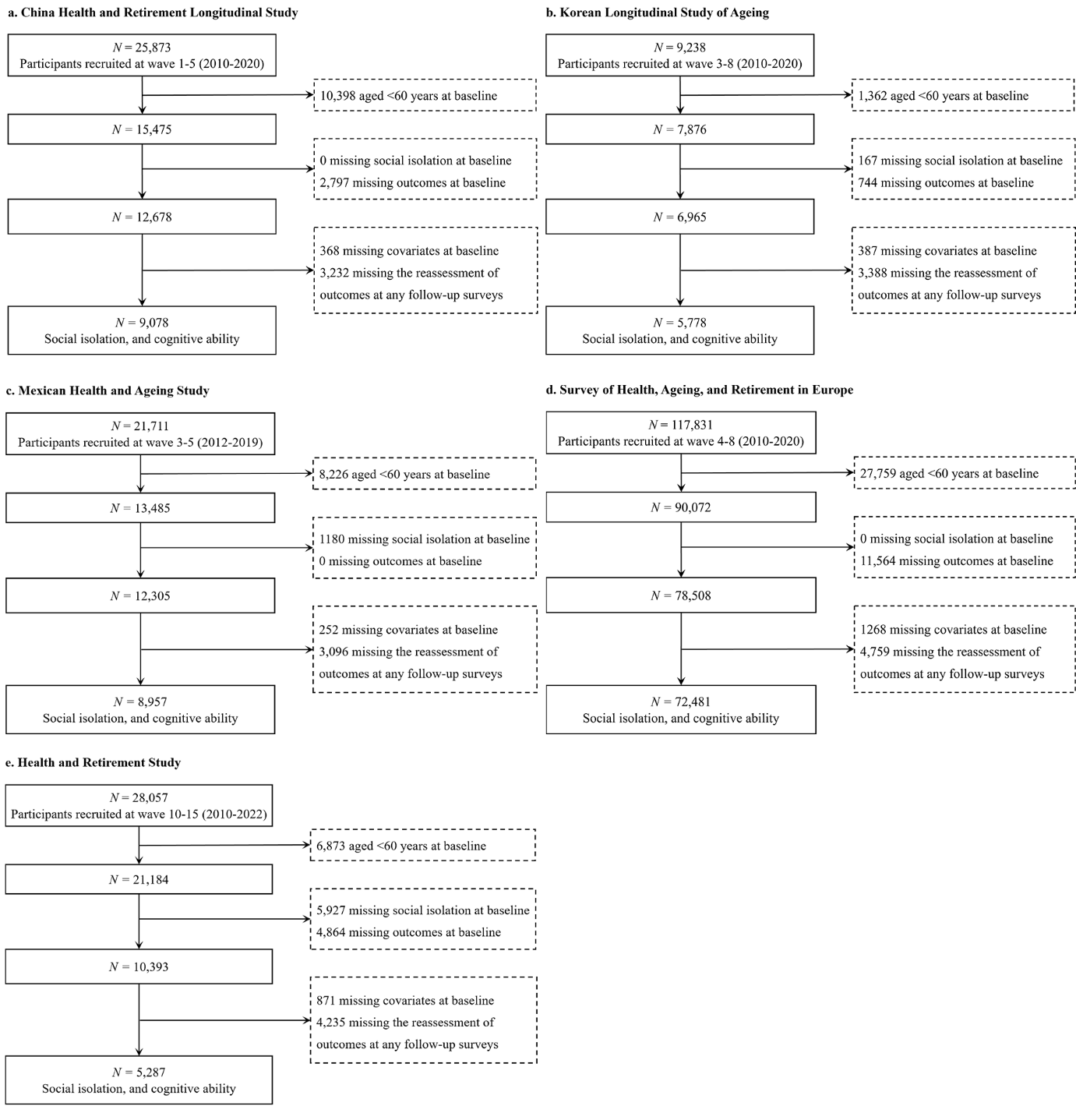
Extended Data Fig.1** Sample selection flow chart of each longitudinal survey data**. a**, China Health and Retirement Longitudinal Study (CHARLS). **b**, Korean Longitudinal Study of Ageing (KLoSA). **c**, Mexican Health and Ageing Study (MHAS). **d**, Survey of Health, Ageing, and Retirement in Europe (SHARE). e, Health and Retirement Study (HRS).

**
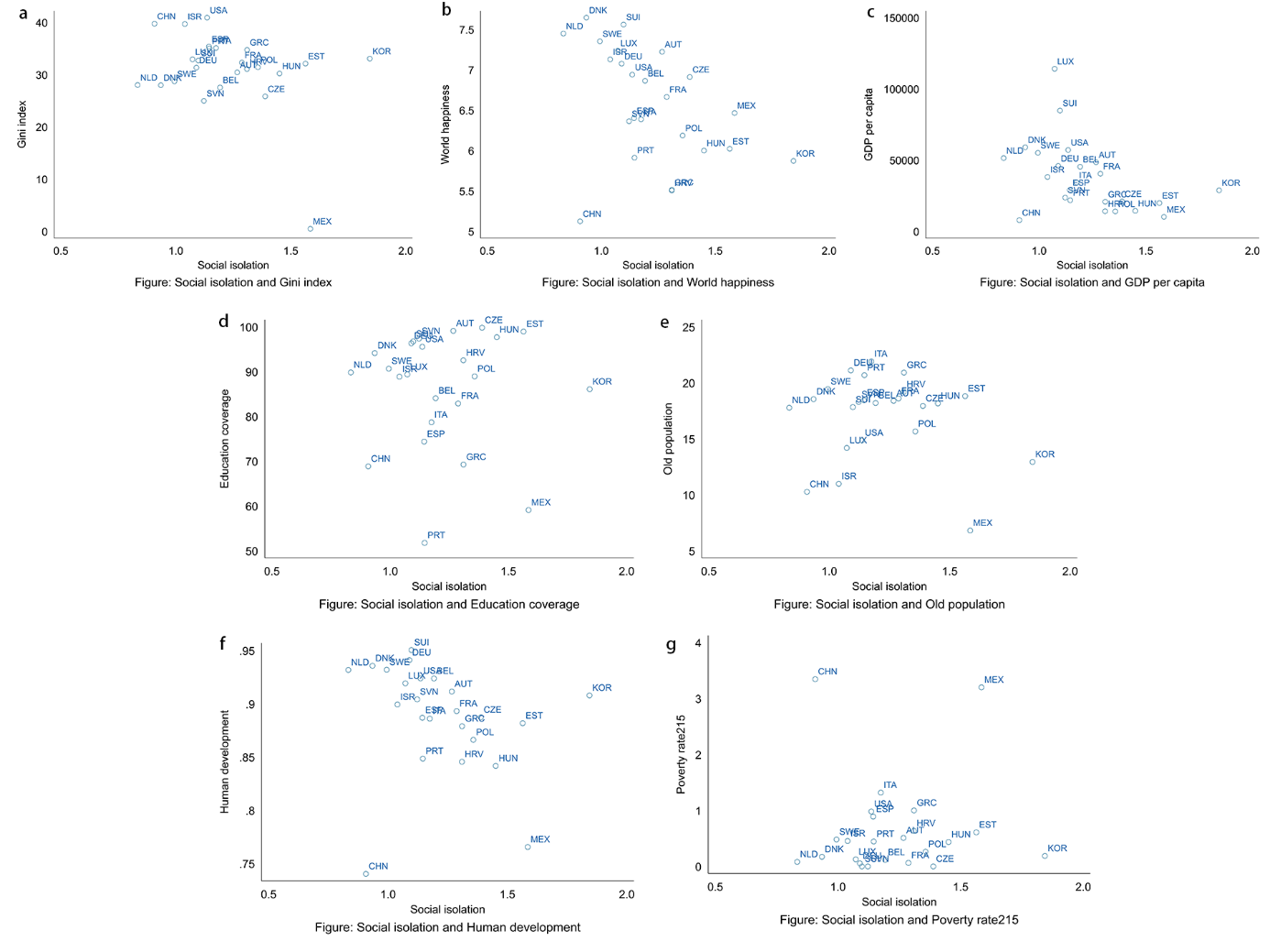
Extended Data Fig. 2** Correlations between social isolation rate and country-level factors**.** **a**, Social isolation and Gini index. **b**, Social isolation and world happiness index. **c**, Social isolation and GDP per capita. **d**, Social isolation and education coverage. **e**, Social isolation and old population. **f**, Social isolation and human development index. **g**, Social isolation and poverty rate. Data are presented as mean values. AUT, Austria; BEL, Belgium; CHN, China; HRV, Croatia; CZE, Czech Republic; DNK, Denmark; EST, Estonia; FRA, France; DEU, Germany; GRC, Greece; HUN, Hungary; ISR, Israel; ITA, Italy; KOR, Korea; LUX, Luxembourg; MEX, Mexico; NLD, Netherlands; POL, Poland; PRT, Portugal; SVN, Slovenia; ESP, Spain; SWE, Sweden; SUI, Switzerland; USA, United States.

**
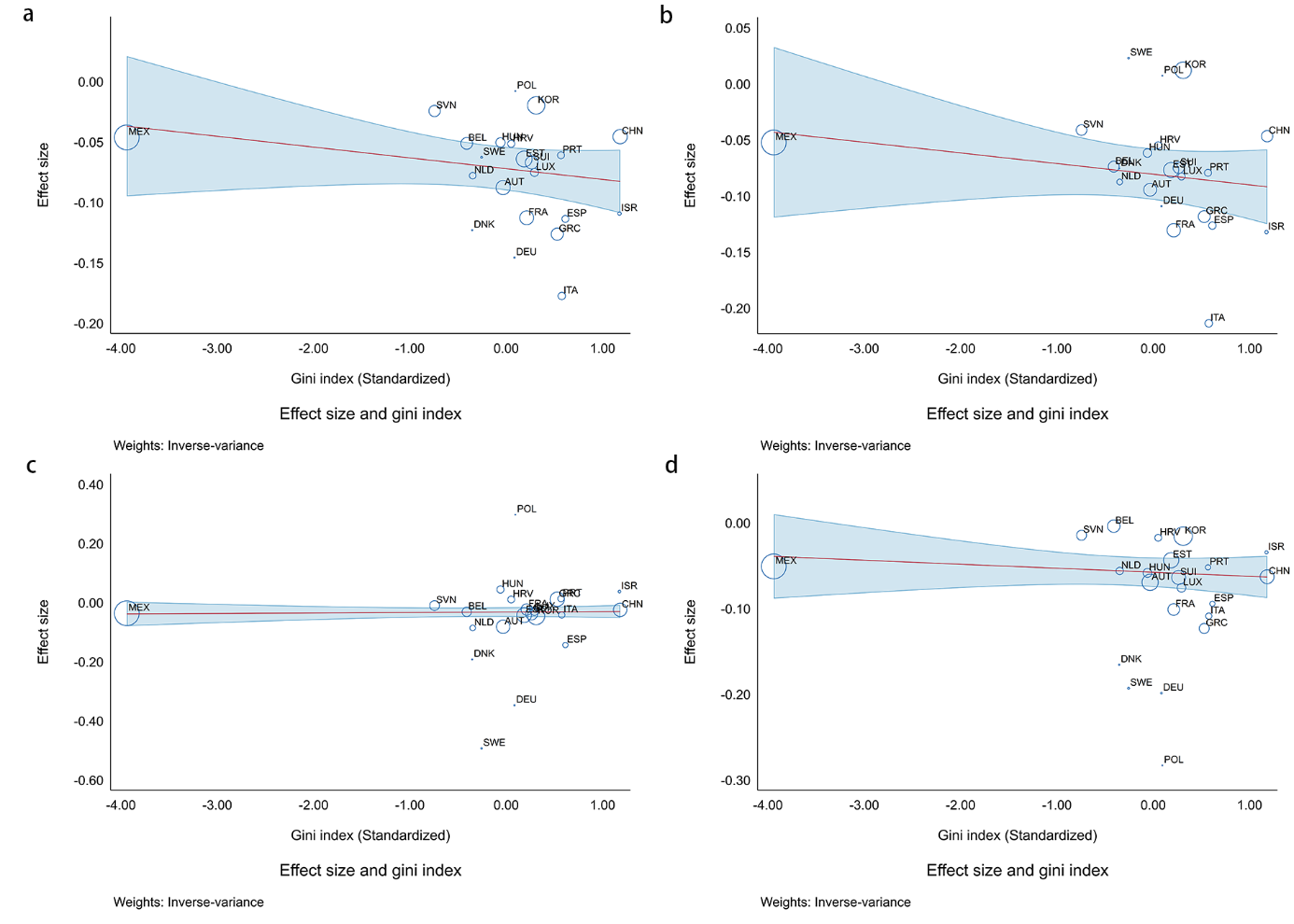
Extended Data Fig.3** Bubble plot with fitted meta regression line of the effect size for four outcomes and Gini index. Data are presented as linear mixed effects coefficients (bubbles), 95% confidence intervals (shaded area) and the linear prediction (red line). **a**, Cognitive ability. **b**, Memory ability. **c**, Orientation ability. **d**, Executive ability. AUT, Austria; BEL, Belgium; CHN, China; HRV, Croatia; CZE, Czech Republic; DNK, Denmark; EST, Estonia; FRA, France; DEU, Germany; GRC, Greece; HUN, Hungary; ISR, Israel; ITA, Italy; KOR, Korea; LUX, Luxembourg; MEX, Mexico; NLD, Netherlands; POL, Poland; PRT, Portugal; SVN, Slovenia; ESP, Spain; SWE, Sweden; SUI, Switzerland; USA, United States. The same applies to the in-plot detail in Extended Figure 4-6.

**
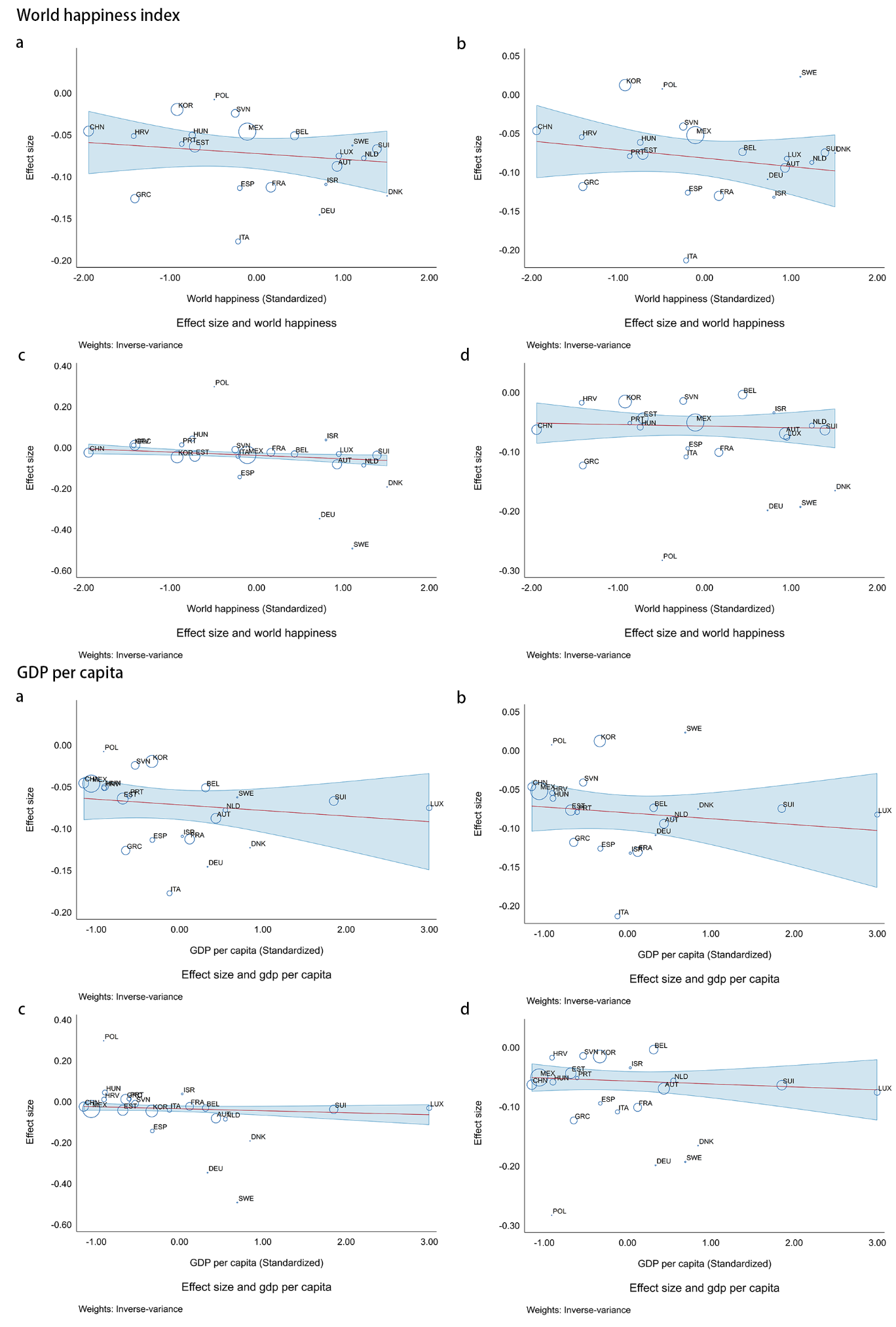
Extended Data Fig.4** Bubble plot with fitted meta regression line of the effect size for four outcomes and world happiness index and GDP per capita.

**
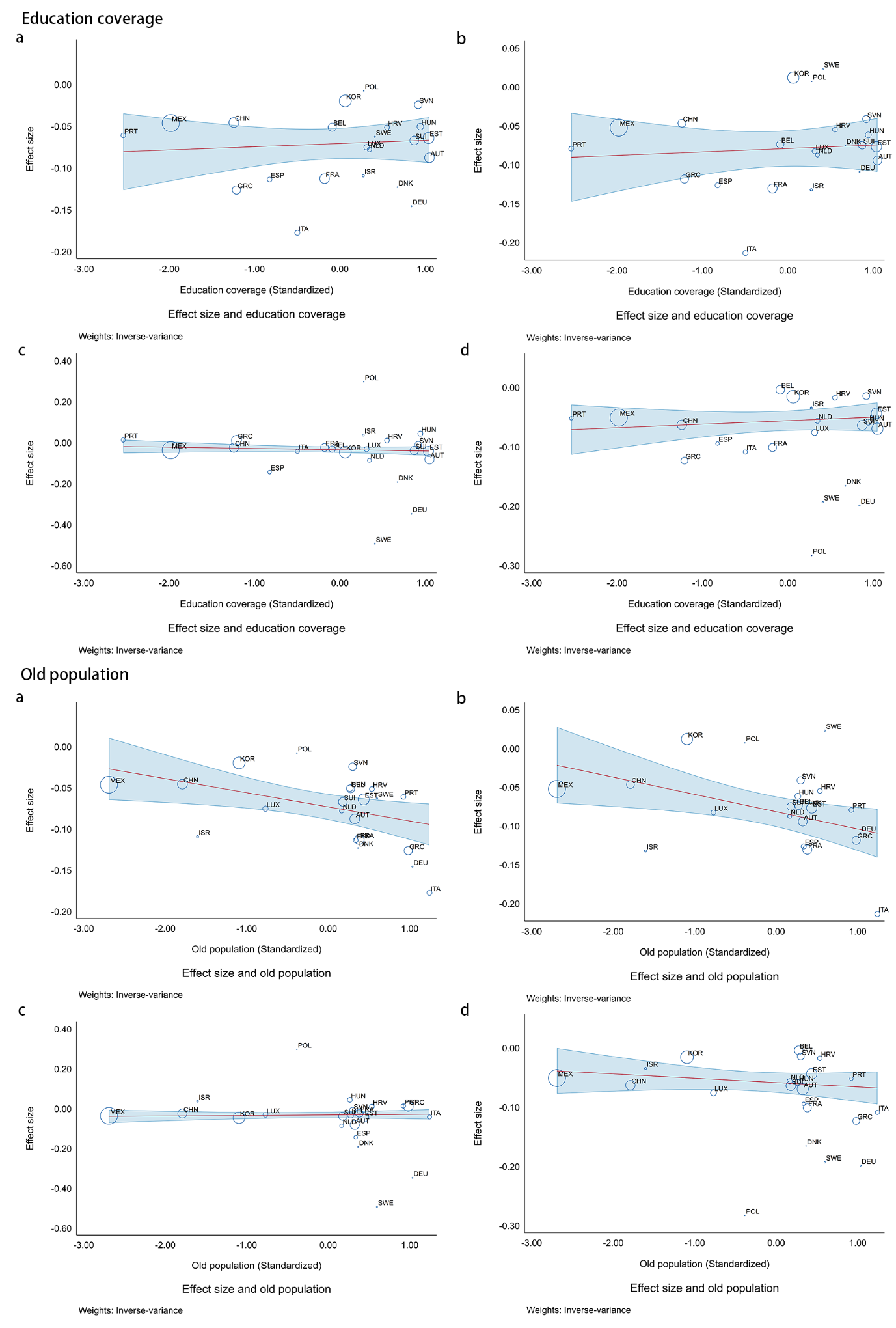
Extended Data Fig.5** Bubble plot with fitted meta regression line of the effect size for four outcomes and education coverage and old population.

**
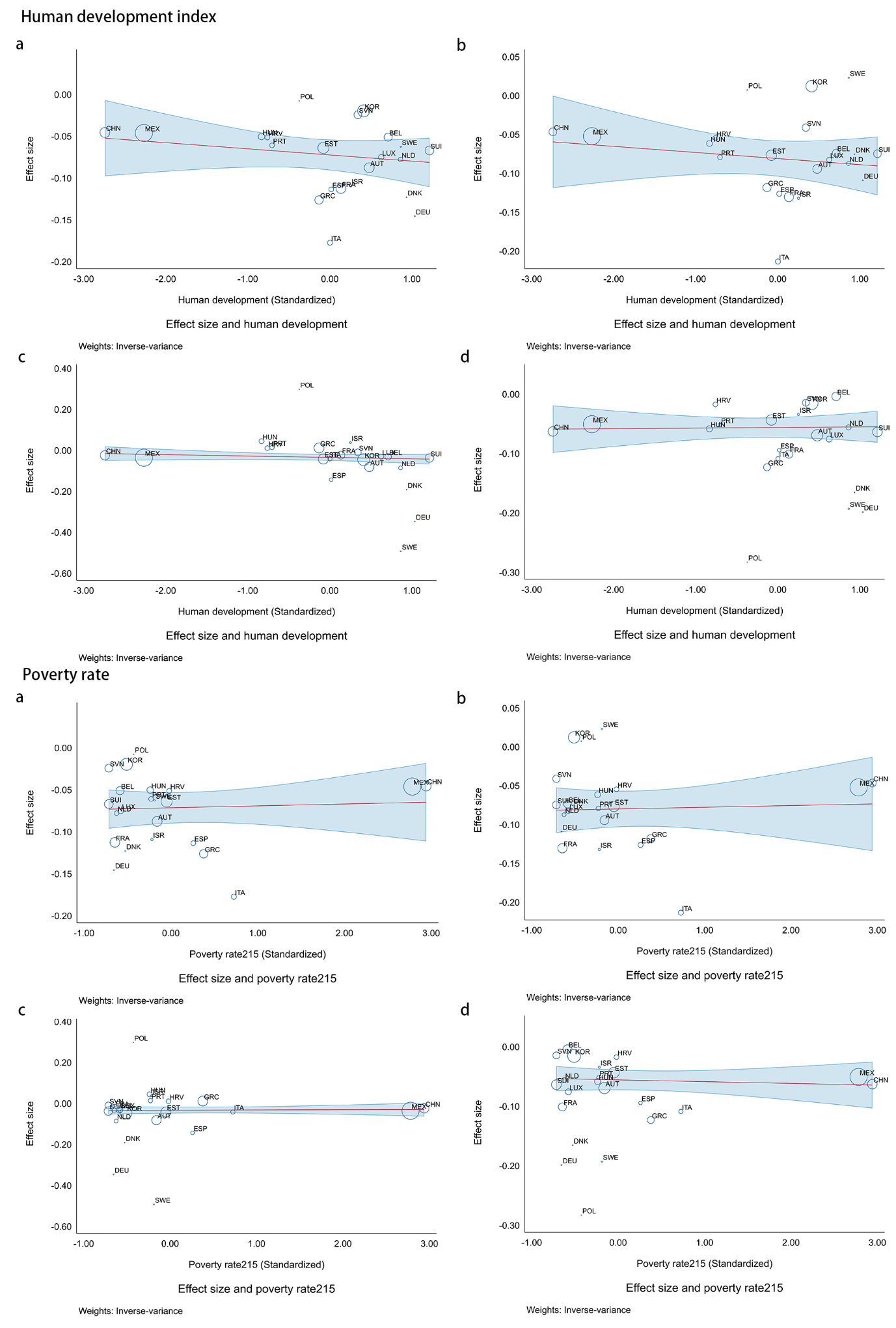
Extended Data Fig.6** Bubble plot with fitted meta regression line of the effect size for four outcomes and human development index and poverty rate.


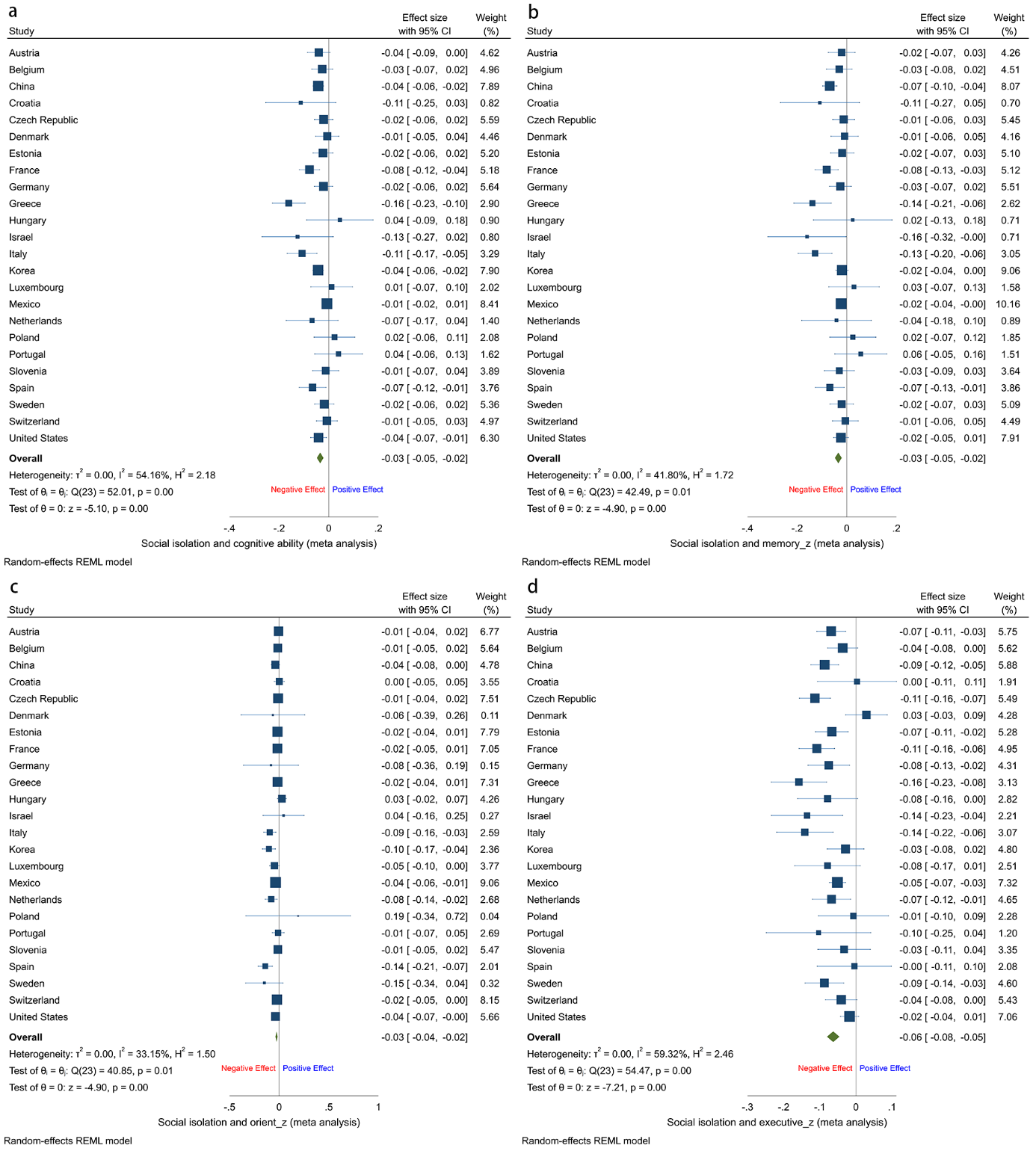
**Extended Data Fig.7** Sensitivity analysis results of changing the baseline model to a fixed-effects model. **a**, Cognitive ability. **b**, Memory ability. **c**, Orientation ability. **d**, Executive ability.

**
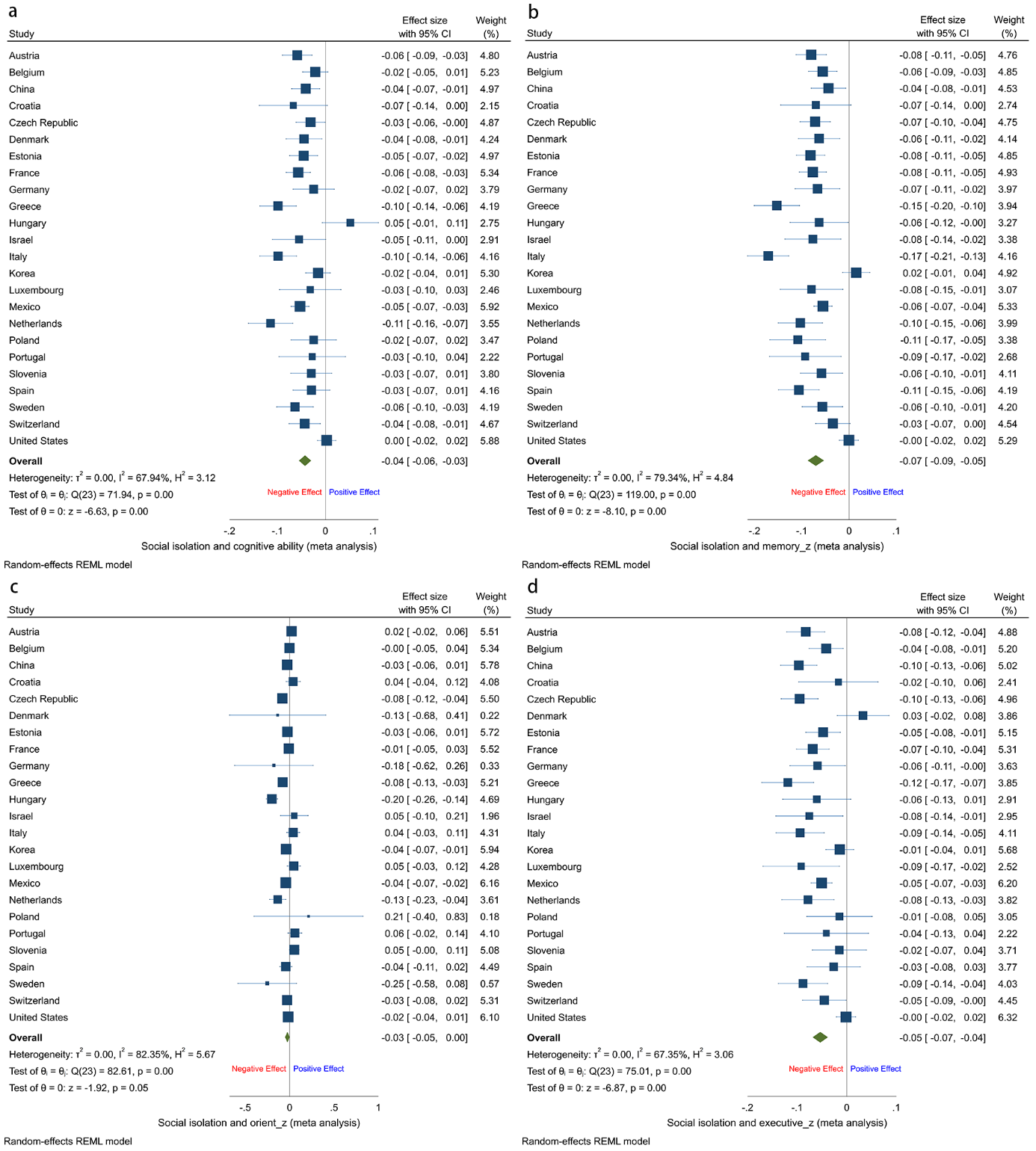
Extended Data Fig.8** Sensitivity analysis results of using multiple interpolation for missing data. **a**, Cognitive ability. **b**, Memory ability. **c**, Orientation ability. **d**, Executive ability.

**
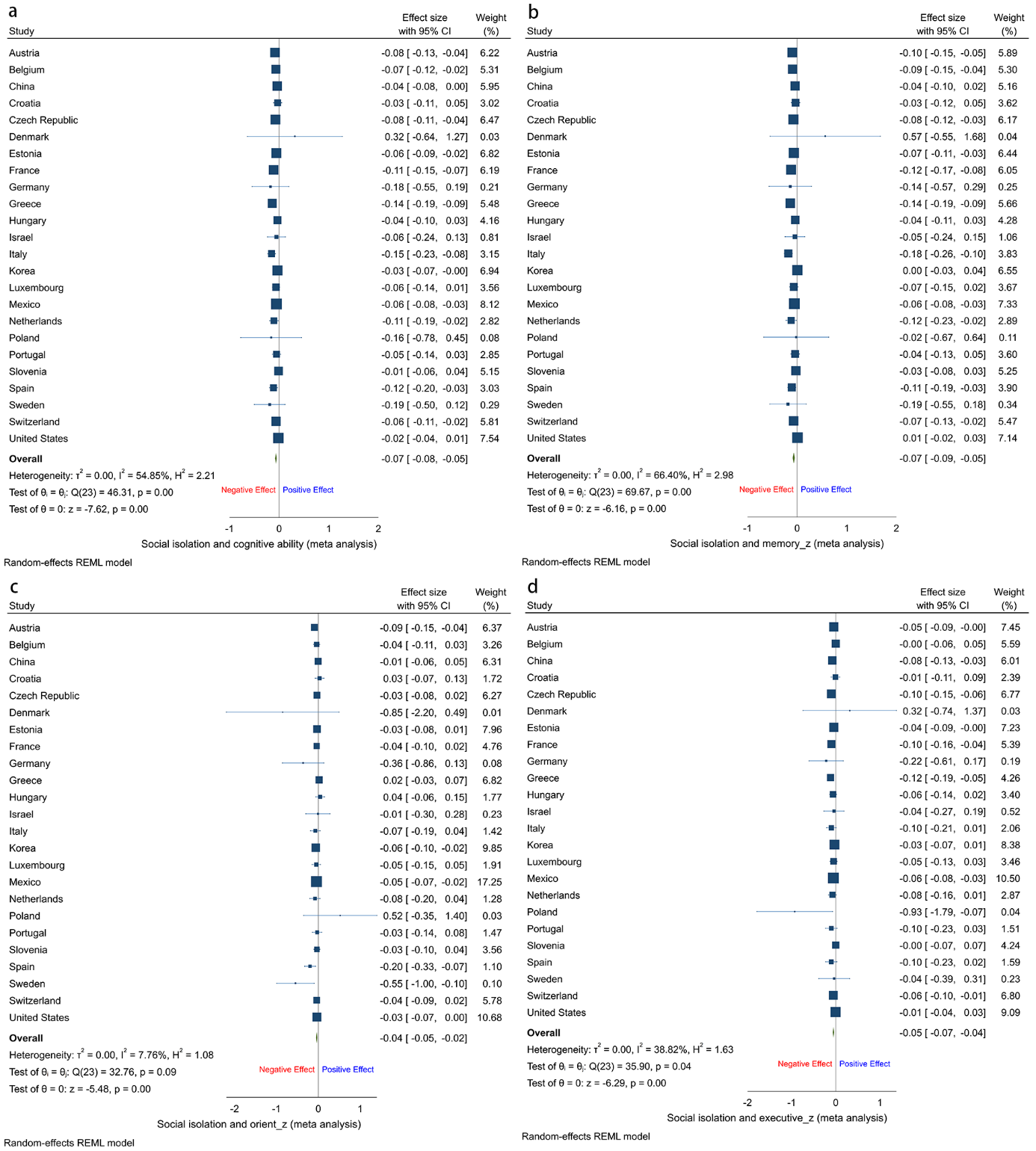
Extended Data Fig.9** Sensitivity analysis results of defining the age of older adults as 65 years and over. **a**, Cognitive ability. **b**, Memory ability. **c**, Orientation ability. **d**, Executive ability.
